# Cout direct hospitalier des accidents vasculaires cérébraux à Parakou au nord du Benin

**DOI:** 10.11604/pamj.2013.16.121.2790

**Published:** 2013-11-28

**Authors:** Thierry Adoukonou, Philomène Kouna-Ndouongo, Jean-Mannix Codjia, Richmine Covi, Francis Tognon-Tchegnonsi, Pierre-Marie Preux, Dismand Houinato

**Affiliations:** 1 UER Neurologie, Faculté de Médecine Université de Parakou, 03BP10 Parakou, Benin; 2 Unité de Neurologie, Centre Hospitalier départemental du Borgou, Parakou, BP 02 Parakou, Benin; 3 Département de Neurologie, Université des Sciences de la Santé, Libreville, GABON; 4 UER psychiatrie Faculté de médecine Université de Parakou, Benin; 5 INSERM UMR1094, Neuroépidémiologie Tropicale, Limoges, France; Université de Limoges, Faculté de Médecine, Institut d'Epidémiologie neurologique et de Neurologie Tropicale, CNRS FR 3503 GEIST, Limoges, France; CHU Limoges, France; 6 UER Neurologie, Faculté des Science de la Santé, Université d'Abomey-Calavi, Bénin

**Keywords:** Cout, cout direct, accident vasculaire cérébral, Cost, direct cost, stroke

## Abstract

**Introduction:**

Les accidents vasculaires cérébraux constituent un véritable problème de santé publique en Afrique avec une charge importante. Les données fiables sur sa réelle charge économique sont rares en Afrique. L'objectif de cette étude était d’évaluer le coût direct hospitalier des AVC à Parakou au Bénin.

**Méthodes:**

Il s'agissait d'une étude transversale économique ayant inclus des patients hospitalisés pour un AVC à l'hôpital de Parakou entre le 1^er^ Juin 2010 au 31Mai 2011. Les données concernant les différents postes de consommation ont été collectées selon la méthode dite bottom-up. Le coût était envisagé du point de vue de la société et du patient. L'unité du coût était le franc CFA (valeur en 2011). Une régression linéaire multiple était utilisée pour déterminer les meilleurs prédicteurs du coût.

**Résultats:**

Ils étaient 78 patients dont 52 hommes, âgés en moyenne de 57 ans ± 10.9. Le NIHSS moyen était de 14,4. Le taux de mortalité était de 20,5%. Le coût direct moyen était de 316.810,3 (±230.774,8) F CFA (environ 704 ± 512 Euros). Les grands postes de consommation étaient les explorations paracliniques (34.3%) les soins et médicaments (28.4%) et les frais d'hospitalisation (17.9%). Les meilleurs prédicteurs du coût élevé étaient un AVC hémorragique, un NIHSS élevé à l'admission et une longue durée d'hospitalisation.

**Conclusion:**

Cette étude suggère un coût élevé de la prise en charge actuelle des AVC à Parakou.

## Introduction

Les accidents vasculaires cérébraux (AVC) constituent en Afrique sub-saharienne un problème majeur de santé publique de part leur incidence et leur morbimortalité [1]. La situation actuelle du continent caractérisée par un manque de ressources humaines et matérielles dans la prise en charge des AVC ne laisse guère la place à l'optimisme [2]. La prévalence des AVC au Bénin est estimée dans une étude récente à 460 pour 100.000 habitants à Cotonou [3] et ils constituent la première cause d'hospitalisation et de décès dans le service de neurologie à Cotonou [4].

L'absence de système de sécurité sociale, le délai tardif de prise en charge des patients et le défaut de dépistage des facteurs de risque vasculaire viennent assombrir le tableau. Actuellement le paludisme, la tuberculose et le VIH-SIDA constituent toujours de réels défis pour les politiques de santé et de développement en Afrique. L'Afrique est à la croisée des chemins. Faire des choix stratégiques et avoir une vision prospective et intégrée de tous les problèmes de santé: faire face aux fléaux actuels et prévenir l’épidémie de demain. Pour permettre aux politiques d'opérer des choix rationnels il faut avoir des données fiables sur le coût et la charge réelle des différentes affections. Ainsi les études sur le coût des AVC sont indispensables pour donner une idée de la charge économique de la maladie. Au Sénégal une étude a estimé ce coût hospitalier à 78426 FCFA par patient [5] tandis qu'au Togo le coût moyen direct d'un AVC par patient était estimé à 387452,6 FCFA [6]. Ces études bien que donnant une idée du coût souffrent de quelques écueils méthodologiques. Au Bénin aucune donnée fiable n'est disponible sur le coût direct hospitalier des AVC.

L'objectif de cette étude préliminaire était de déterminer le coût direct hospitalier des AVC à Parakou au Nord du Bénin ainsi que des facteurs qui lui sont associés.

## Méthodes

### Cadre d’étude

L’étude s'est déroulée dans le centre hospitalier départemental du Borgou à Parakou. Il s'agit du plus grand hôpital dans le septentrion béninois et situé à 420 Km environ de Cotonou. Depuis la création de l'université de Parakou avec sa faculté de médecine, il est devenu un centre universitaire. La ville de Parakou dispose d'une unité de scanner située dans un centre confessionnel privé à moins de 1Km de l'hôpital et offre aussi des facilité dans les explorations cardiovasculaires (Electrocardiogramme, Echodoppler des vaisseaux du cou et Echographie cardiaque) à l'hôpital d'instruction des armées. Les patients ont été recrutés dans les services d'urgence, de réanimation de médecine et de neurologie dudit hôpital.

### Méthodes d’étude

**Type et période d’étude** Il s'est agi d'une étude prospective de type économique, à visée descriptive et analytique qui s'est déroulée du 1^er^ Juin 2010 au 31 Mai 2011.

**Population d’étude**: Elle est constituée de l'ensemble des sujets victimes d'accident vasculaire cérébral et hospitalisé au CHD Borgou quel qu'en soit le devenir.

**Echantillonnage:** Un recrutement systématique et exhaustif des patients ayant rempli les critères d'inclusion et accepté de participer à l’étude a été effectué.

**Critères d'inclusion:** Etre hospitalisé pendant la période d’étude dans l'une des deux structures pour un accident vasculaire cérébral défini suivant les critères de l'OMS [7]. Avoir donné son consentement (ou à défaut celui d'un proche parent) pour participer à l’étude.

**Critères d'exclusion:** Tout patient ayant une hémorragie méningée, une thrombose veineuse cérébrale ou un déficit neurologique en rapport avec un traumatisme crânien ou une tumeur cérébrale ou autre cause.

**Variable dépendante:** il s'agit du coût hospitalier direct qui est la somme des coûts par catégorie (consultations, transports, traitement, examens paracliniques, rééducation, frais d'hospitalisation, autres frais pendant hospitalisation) ainsi que la valeur monétaire du temps passé par l'accompagnant principal.

**Variables indépendantes:** sociodémographiques (âge, sexe, ethnie, profession), cliniques (score neurologique NIHSS à l'admission, type d'AVC, délai d'admission), hospitalières (durée d'hospitalisation, service d'hospitalisation), pronostiques et fonctionnelles (RANKIN à la sortie, BARTHEL, NIHSS à la sortie, mode de sortie), et étiologiques (type d'AVC ischémique, d'AVC hémorragique).

**Collecte de données:** Le recueil des données a été effectué de façon prospective. Les patients ont été inclus dès leur admission à l'hôpital. Une fiche standardisée a servi au recueil des données. Les dépenses relatives aux différents postes de consommation étaient systématiquement relevées au fur et à mesure, tous les jours y compris les week-ends et jours fériés. Les soins et actes divers non payés ont été valorisés par leur coût réel.

**Approche économique:** La technique de recueil adoptée a été l'approche Bottom-up. Il s'agit de l'approche individuelle sur les dépenses relatives à chaque patient à travers un questionnaire où systématiquement sont recensées toutes les dépenses. L’évaluation du coût a été faite du point de vue sociétal et du point de vue du patient. La période d'estimation du coût était la période hospitalière.

### Analyse statistique

Toutes les données collectées ont été saisies et analysées par les logiciels Epi-info version 6.04d. et SPSS (Statistical Package for Social Sciences) version 8. Les variables quantitatives ont été exprimées en moyenne avec leur écart-type, les données qualitatives en pourcentage et leur intervalle de confiance à 95%.

La comparaison des variables qualitatives a été faite en utilisant le test de chi-2 (ou le test exact de Fisher selon le cas) et celle des moyennes par le test de Student, Mann-Whitney ou de Wilcoxon selon le cas.

Pour l'analyse multivariée une régression linéaire multiple a été utilisée en procédant à des itérations successives de type pas à pas descendant et en y introduisant toutes les variables significativement associées au coût en analyse univariée. Nous avons ainsi écrit et déterminé un modèle final de l’équation avec le coefficient Bêta, l'erreur standard ainsi que la valeur de p. Cette équation pouvant s’écrire C = β_0_+Σβ_j_X_j_+ɛ - X_j_ étant des covariables d'intérêt et C le coût, β_0_ la constante ou l'intercept de la droite.

Un p < 0.05 a été considéré comme statistiquement significatif.

### Considérations éthiques

Le projet a été soumis aux autorités de l'hôpital et obtenu l'approbation du directeur. Un consentement éclairé et écrit de chaque patient et ou de son entourage était obtenu après avoir expliqué le but et les modalités de l’étude.

## Résultats

Au total 78 patients ont été inclus avec un âge de 36 à 80 ans (moyenne 57.0 ans ±10.9 ans) et comportant 52 hommes (66.7%). Les principaux facteurs de risque vasculaire identifiés chez les patients étaient l'hypertension artérielle (71.8%), le diabète (12.8%) le tabagisme (5.1%). Le score neurologique (NIHSS) à l'admission allait de 4 à 30 avec une moyenne de 14.4 (±5.3). Trente quatre patients (43.6%) avaient un NIHSS supérieur à 15. L’état neurologique (NIHSS) des patients était identique à l'admission dans les différents services, 15.5 (±4.7) pour le service de réanimation et 13.9 (±5.6) pour le service de neurologie (p = 0.216).

Parmi les 78 patients, 69 (88.5%) ont pu réaliser un scanner cérébral. Les types d'AVC se répartissaient ainsi: AVC ischémique: 36 sujets (46.2%), AVC hémorragique: 33 sujets (42.3%), AVC indéterminé (n'ayant pu faire le scanner cérébral): 9 sujets (11.5%). La durée d'hospitalisation variait de 2 à 46 jours avec une moyenne de 14.4 jours (±10.1). Le mode de sortie était un retour à domicile pour 50 patients (64.1%), 11 étaient sortis contre avis médical (14.1%) et 17 (21.8%) étaient décédés. Parmi les 61 patients vivant à la sortie 34 (55.7%) étaient indépendants à la sortie (RANKIN ≤ 2). Le taux de mortalité était plus élevé en réanimation 50,0% (12/24) qu'en neurologie 7.7% (4/52) (p < 10^−4^). Pendant la période d'hospitalisation, la dépense totale en coût direct variait de 38.500F à 1.099.700F avec une moyenne de 316.810,3F (±230774,8), la médiane étant de 262.565F. Le Table 1 résume la répartition du coût suivant les postes de consommation.

**Table 1:** Répartition du coût direct hospitalier des AVC par poste de consommation.

	Moyenne (+/− écart-type)	[Min – Maxi]	% coût total
**Transport**	39.070,5 (+/ − 41.410,1)	[2.000 – 175.000]	13.7
**Consultation**	6.114,1 (+/ − 5.244,8)	[0 – 12.000]	2.1
**Examens paracliniques**	97.990,4 (+/ − 78875)	[9.500 – 194.300]	34.3
**Hospitalisation**	50.969,1 (+/ − 77.280,4)	[0 – 550.000]	17.9
**Soins et médicaments**	80.993,1 (+/ − 60.408,5)	[4.500 – 382.350]	28.4
**Kinésithérapie**	10.384,6 (+/ − 19.963,1)	[0 – 90.000]	3.6

Tous les patients avaient un accompagnant principal. Le coût du temps passé par les accompagnants variait de 15000F à 150000F avec une moyenne de 43397,4F (±23665,6F) et une médiane de 35000F.

L’âge et l'ethnie étaient associés au coût élevé des AVC tandis que le sexe et la profession n’étaient pas associés. Ces données sont résumées dans le Table 2.

**Table 2:** Coût direct hospitalier des AVC suivant les données sociodémographiques, Parakou, 2011

	Moyenne (+/− Ecart-type)	p
**Age (ans)**		**0.034**
35 – 44	371945.6 (+/ − 330085.8)	
45 – 54	230807.6 (+/ − 129422.3)	
55 – 64	273583.6 (+/ − 184487.7)	
65 – 74	432679.2 (+/ − 247926.1)	
75 – 80	449585.0 (+/ − 408648.9)	
**Sexe**		**0.834**
Masculin	320711.1 (+/ − 206116.9)	
Féminin	309008.7 (+/ − 277972. 8)	
Ethnie		0.015
Fon et apparentés	388244.6(+/ − 264168.9)	
Nagots et apparentés	391036.3 (+/ − 263242.7)	
Baribas	188063.8 (+/ − 75469.2)	
Dendis	132518.8 (+/ − 85133.8)	
Minas	306284.3 (+/ − 138725.9)	
Peulhs	186351.7 (+/ − 107928.9)	
**Profession**		**0,111**
Ménagère	246351.9 (+/ − 136578.3)	
Cultivateurs	102370.0 (+/ − 61651.1)	
Revendeuses	346100.0 (+/ − 325926.1)	
Cadres et assimilés	354856.7 (+/ − 175238.6)	
Artisans, ouvriers et autres	362339.7 (+/ − 304923.7)	

Sur le plan clinique et fonctionnel la durée d'hospitalisation le score neurologique à l'admission, le type d'AVC et le RANKIN à la sortie étaient les facteurs influençant le coût. Le Table 3 résume ces données. Le service d'hospitalisation n'influençait pas le coût. En analyse multivariée utilisant la régression linéaire multiple l’équation du modèle final s’écrit comme résumée dans le Table 4.

**Table 3:** Coût direct hospitalier des AVC suivant les données cliniques

	Moyenne (+/− Ecart-type)	p
**NIHSS à l'admission**		0,009
4 - 9	184678.9 (+/ − 202119.7)	
10 - 14	261793.1 (+/ − 148221.9)	
15 - 19	376275.0 (+/ − 283421.6)	
20 - 24	408639.4 (+/ − 277807.5	
25 - 30	576700,0 (+/ − 199696.7)	
**Type AVC**		0,002
AVC ischémique	325962.8 (+/ − 204412.3)	
AVC hémorragique	373087.1 (+/ − 249348.1)	
AVC indéterminé	73851.7 (+/ − 35288.7)	
**Durée d'hospitalisation**		0,0001
2-9	163406.4 (+/ − 115928.8)	
10-19	335948.3 (+/ − 180470.1)	
20-29	349126.3 (+/ − 148405.0)	
>macr;30	645541.4 (+/ − 271164.8)	
**RANKIN à la sortie**		0.040
1	277311.7 (+/ − 184110.0)	
2	291920.9 (+/ − 211298.1)	
3	300251.7 (+/ − 232913.6)	
4	665691.3 (+/ − 344711.9)	
6 (décès)	312058.2 (+/ − 202426.1)	

**Table 4:** Modèle final de l’équation en régression linéaire, des facteurs associés au coût direct hospitalier des AVC

	Erreur standard	Coefficient Bêta	p
Constante	0.166		0.0001
Type d'AVC (hémorragique)	0.069	−0.407	0.0001
NIHSS (plus élevé)	0.009	0.330	0.0001
Durée d'hospitalisation (plus longue)	0.005	0.570	0.0001

## Discussion

Le principal objectif de cette étude était de déterminer le coût direct hospitalier des AVC à Parakou. Il s'agit à notre connaissance de la première étude conduite sur l’évaluation du coût direct des AVC en période hospitalière au Bénin. Il s'agit d'une étude du coût de la maladie (COI/ Cost Of Illness) qui donne des éléments descriptifs généraux sur la pathologie. Nous avons, dans notre approche méthodologique, défini la pathologie (AVC), la période d'estimation du coût (hospitalière), le type de coût (direct), le mode de collecte des données (bottom-up method), la valorisation des volumes collectés. Cette approche méthodologique répond aux exigences des études économiques [8]. Le coût des AVC estimé ici l'est du point de vue général car tient à la fois compte de ce que paient le patient et la société. En effet lorsque des patients étaient pris en charge par des assurances (privées ou publiques) les frais réels des actes et soins étaient comptabilisés.

Le coût des AVC dans cette étude était de 316.810,3F. Ce coût rapporté au produit national brut par habitant du Bénin en 2011 était important et représentait 1.03 fois le PNB/habitant. Les postes de consommation les plus importants étaient les examens paracliniques et les soins avec respectivement 34.3% et 28.4% expliquant à eux seuls plus de 60% de la variance du coût. Les comparaisons des coûts dans les différentes études ne peuvent se faire directement car ce coût dépend de plusieurs facteurs liés surtout au système sanitaire de chaque pays et doit prendre en compte le point de vue envisagé (patient = out of pocket; l’état, les assurances, la société), le type de coût et la période du coût. Toutefois quelques études réalisées sur le coût direct des AVC permettent de donner une idée de la charge de la pathologie dans quelques pays. LeTable 5 résume des données existantes sur le coût direct des AVC.

**Table 5:** Le coût direct des AVC dans quelques études

Pays [références]	Année/Type de coût	Type d’étude	Taille /population	Type AVC	Période	Coût
**Grèce** [9]	2001 / Direct	Prospective	429/hospitalière	AIC/AVCH	Aiguë	3624.9 euros/patient
**Singapour** [10]	2002 / Direct	Prospective	200 / hospitalière	Tous (OMS)	Aiguë	2410.83 dollars /patient
**France** [11]	2004 / Direct	Prospective	454 / hospitalière	AIC/AVCH	1 an	17 799 euros/patient
**Canada** [12]	1994 / Direct	Prospective	285 / hospitalière	AIC/AVCH	Aiguë	17328 euros/patient
**Suède** [13]	1994/ Direct	Prospective)	287 / hospitalière	AIC/AVCH)	Aiguë/réhabilitation	1.3 milliard $ US
**Taiwan** [14]	2003/ Direct	Prospective	360 / hospitalière	AIC Aigue	Aiguë	841 $ US / patient
**Japon** [15]	2003/ Direct	Prospective	179 / hospitalière	AIC	Aiguë	6887 $ US / patient
**USA** [16]	1996 / Direct	Prospective	191 / hospitalière	AIC	Aiguë	4408 $ US / patient
**Grande-Bretagne** [17]	2005/ Direct/indirect	Prospective	Incidence/prévalence	AVC	1 an	9 milliard £ /an
**Allemagne** [18]	2005 /Direct	Prospective	383 / hospitalière	AVC et AIT	1 an	9452 euros
**Italie** [19]	2005 /Direct/Indirect	Prospective	449/hospitalière, Incidence	AVC (OMS)	3, 6,12mois	11600 euros/patient
**Sénégal** [5] **Togo** [6] **Bénin**	1997 /Direct 1997/Direct 2011 /Direct	Rétrospective Prospective Prospective	383 / hospitalisés 104/hospitalisés 78 /hospitalisés	AVC AVC AIC/AVCH	Aiguë Aiguë Aiguë	78426 FCFA / patient 387453FCFA/patient 316810FCFA/ patient

AVC: Accident vasculaire cérébral, AIC: Accident ischémique cérébral, AVCH: accident vasculaire cérébral hémorragique

Ce coût bien que faible est comparable à celui obtenu dans plusieurs études africaines. En effet au Togo [6] dans une étude portant sur 104 patients les auteurs ont rapporté un coût de 387453FCFA/patient. Au Sénégal par contre le coût par patient était de 78426 FCFA / patient [5]. L’équipe sénégalaise a utilisé la méthode top-down en collectant des données à partir d'une base de données. Le système sanitaire sénégalais n’étant pas très différent du nôtre où chaque patient devrait payer ses soins, nous pensons que cette stratégie n’était peut-être pas adaptée car elle ne prend nullement en compte les dépenses effectuées par le patient. Nous pensons qu'il est quasi impossible de dissocier la consommation de ressources de l'hôpital comme l'eau ou l’électricité en rapport avec les AVC de celle en rapport avec les autres pathologies pendant la période hospitalière. Les frais d'hospitalisation dans notre contexte prennent en compte la restauration et l'hôtellerie. La durée d'hospitalisation aussi était un important facteur associé au coût. En effet les patients ayant un séjour plus long étaient ceux qui avaient un coût élevé. La durée d'hospitalisation étant d'une part en rapport avec la survenue de complications et d'autre part en rapport avec le déficit neurologique la prise en charge de ces complications pourraient engendrer des coûts supplémentaires. C'est le déterminant majeur du coût à la phase aigue des AVC. Ceci est en accord avec la plupart des données de la littérature [9, 10, 18].

Le type d'accident vasculaire cérébral était également associé au coût direct. En effet les sujets ayant un AVC hémorragique avaient le coût le plus élevé. Cette tendance a été confirmée par une étude américaine sur l’évaluation du coût sur la vie entière [10]. Les AVC hémorragiques à la phase aiguë étant cliniquement plus sévères, le coût serait plus élevé à cette période mais les coûts des explorations dans les infarctus cérébraux étant plus importants, le coût sur une longue période serait plus important chez ces derniers. Par ailleurs les AVC hémorragiques ont un meilleur pronostic fonctionnel que les infarctus, le coût sur la vie entière devrait être plus important dans les infarctus. Nos résultats peuvent s'expliquer par la période d'estimation du coût, car il est seulement évalué sur la période hospitalière et aussi par le fait que beaucoup d'infarctus n'avaient pas bénéficié d'explorations appropriées à visée étiologique. Le service d'hospitalisation n'a pas influencé le coût. Mais d'un point de vue pronostique la mortalité dans le service de réanimation était de 50% et plus élevé que celui observé en neurologie 7.7% alors que l’état neurologique à l'admission dans les deux services était identique. Nous postulons que l'organisation de la prise en charge dans les deux services pourrait expliquer cette différence. En effet l'unité de neurologie est dirigée par un neurologue, spécialiste en pathologie neurovasculaire. Dans ce service les protocoles de prise en charge suivant les recommandations étaient strictement respectées. On sait que la prise en charge dans des unités neurovasculaires réduit la mortalité par rapport à la prise en charge dans d'autres unités [16].

## Conclusion

Les résultats de cette étude devraient être interprétés avec beaucoup de précaution. Il existe des limites liées à la taille de la population et l'absence de certaines investigations complémentaires. Toutefois bien que limitée cette étude donne une idée de ce que dépense le patient à la phase aigue d'un accident vasculaire cérébral au Bénin. Pour réduire la charge de la maladie un système de KIT comprenant la prise en charge des examens complémentaires diagnostiques et les soins en phase aiguë devrait réduire ce coût. Un tel système pourrait aussi permettre une plus grande accessibilité et améliorer la prise en charge. L'accessibilité financière aux soins est un problème récurrent et peut être les réseaux d'aide communautaire pourraient y jouer un rôle capital [21]. Une étude économique de type coût de la maladie (AVC) est faisable et possible dans notre contexte d'exercice avec une méthodologie correcte prenant en compte tous les aspects des études économiques.
